# Trauma system establishment and outcome improvement: a retrospective national cohort study in South Korea

**DOI:** 10.1097/JS9.0000000000000481

**Published:** 2023-05-18

**Authors:** Junsik Kwon, Myeonggyun Lee, Yoon Kim, Jonghwan Moon, Yo Huh, Seoyoung Song, Sora Kim, Jung-in Ko, Kyoungwon Jung

**Affiliations:** a Division of Trauma Surgery, Department of Surgery, Ajou University School of Medicine; b Ajou University Hospital, Gyeonggi South Regional Trauma Center, Suwon; c Department of Health Policy and Management, Seoul National University College of Medicine; d Department of Emergency Medicine; e National Emergency Medical Center, National Medical Center, Seoul, Korea; f Division of Biostatistics, Department of Population Health, New York University Grossman School of Medicine, New York, USA

**Keywords:** mortality, outcome, performance, trauma center, trauma system, wounds and injuries

## Abstract

**Background:**

Trauma is a major cause of mortality, disability, and health care costs worldwide. The establishment of a trauma system is known to solve these problems, but few studies have objectively evaluated the impact of a trauma system on outcomes. Since 2012, South Korea has established a national trauma system based on the implementation of 17 regional trauma centers nationwide and the improvement of the prehospital transfer system. This study aimed to measure the changes in performance and outcome according to the established national trauma system.

**Material and Methods:**

In this national cohort-based, retrospective follow-up observational study, the authors calculated the preventable trauma death rate (PTDR) by conducting a multipanel review of patients who died in 2015, 2017, and 2019. Furthermore, the authors constructed a risk-adjusted mortality prediction model of 4 767 876 patients between 2015 and 2019 using the extended-International Classification of Disease Injury Severity Scores to compare outcomes.

**Results:**

The PTDR was lower in 2019 than in 2015 (15.7 vs. 30.5, *P* <0.001) and 2017 (15.7 vs. 19.9%, *P*<0.001) representing 1247 additional lives saved in 2019 compared to that in 2015. In the risk-adjusted model, total trauma mortality was highest in 2015 at 0.56%, followed by that in 2016 and 2017 (0.50%), 2018 (0.51%), and 2019 (0.48%), revealing a significant decrease in mortality over the years (*P*<0.001 for trend), representing nearly 800 additional lives saved. The number of deaths for more severe patients with a probability of survival less than 0.25 significantly decreased from 81.50% in 2015 to 66.17% in 2019 (*P*<0.001).

**Conclusions:**

The authors observed a significant reduction in the PTDR and risk-adjusted trauma mortality in the 5-year follow-up since 2015 when the national trauma system was established. These findings could serve as a model for low-income and middle-income countries, where trauma systems are not yet established.

## Introduction

Highlights

- This study measured the changes in trauma performance and outcome according to the national trauma system establishment in South Korea.
- We observed a significant reduction in the preventable trauma death rate and risk-adjusted trauma mortality in a 5-year follow-up since 2015 when the national trauma system was established.
- This study finding could serve as a model for lower-income and middle-income countries, where trauma systems are not yet established.

Injury is the leading cause of death in the economically productive age group (<45 years)^1–3^. According to the most recent Global Burden of Disease Study report, injury causes are among the top causes of disability-adjusted life years in adolescents aged 10–24 years^4^. Improving trauma mortality is a major global public health issue, and many studies have shown that establishing an inclusive trauma system can bring about remarkable reductions in trauma-related mortality, disability, and costs^5–10^. However, there has been little interest or investment in improvement of performance and outcome in trauma care compared to those for infectious diseases or other chronic diseases^3,11^.

Although South Korea is known as one of the high-income countries (HICs), its trauma system is not at the same level as that of other HICs. Despite trauma being one of the three major causes of death in South Korea, alongside cancer and cardiovascular diseases, and one of the four major causes of death in the emergency department^2,12^, the preventable trauma death rate (PTDR) exceeded 30% by the 2010s^13–15^, which was similar to that in low-income and middle-income countries (LMICs)^16–20^.

The establishment of a modern emergency medical system started with the enactment of the Emergency Medical Service Act in 1994, and the current system was established in the 2000s in South Korea^21^. Patients with severe trauma are more time-sensitive than other emergency diseases and require a multidisciplinary simultaneous approach for timely resuscitation. However, there was no consideration or designation for specialized institutions like trauma centers that could provide final definitive care to major trauma patients in the emergency medical system, and as a result, an appropriate transfer system for severe trauma patients was not established until the early 2010s in South Korea. To improve these circumstances, the government and the medical society have revised the Emergency Medical Service Act in 2012 to build the master plan that was based on the implementation of 17 regional trauma centers (RTCs) nationwide and the establishment of a prehospital transfer system so that severe patients can be triaged and transferred to RTCs^21,22^.

This study aimed to measure the changes in trauma performance and outcome based on the national trauma system establishment in South Korea. We hypothesized that the system establishment would improve performance and outcome in trauma care nationwide. To confirm this hypothesis, a serial follow-up survey for PTDR was conducted using a multipanel review process, and the risk-adjusted trauma mortality (RATM) model was used to compare trauma outcome year by year owing to the establishment of a trauma system.

## Material and methods

### Study design, data collection, and setting

This nationwide retrospective observational cohort study was approved by the Institutional Review Board (No. AJIRBMED-EXP-20-473). The requirement for written informed consent was waived, and the researchers analyzed only de-identified (anonymized) data in this study. In addition, this cohort study was registered at the Clinical Research Information Service. The Clinical Research Information Service is a registration system for clinical trials (researches) to be conducted in South Korea. It has been established at the Korea Disease Control and Prevention Agency with support from the Ministry of Health and Welfare. It has joined the WHO International Clinical Trials Registry Platform as the 11th member of the Primary Registry. The hyperlink to our specific registration is publicly accessible. We used the South Korea National Emergency Department Information System (NEDIS) data, which includes healthcare-related information registered in real time by nationwide emergency medical institutions (EMIs) based on the Emergency Medical Service Act. The NEDIS was developed in 2003 to evaluate the performance of emergency care systems in South Korea. In 2019, 401 of 402 EMIs registered nationwide data, with approximately 9 000 000 records^12,23^. We extracted the data of patients with trauma with at least one diagnostic code (S and/or T) based on the Korean version of the International Classification of Disease between 2015 and 2019, according to the NEDIS. Herein, the diagnostic code S indicates different types of injuries in single body regions, and the code T implies injuries in multiple or unspecified body regions as well as poisoning and certain other consequences of external causes^24^. To evaluate the effectiveness of the established trauma system, we compared the PTDR and RATM after 2015, when the trauma system began to operate in earnest. This study has been reported in line with the Strengthening the reporting of cohort, cross-sectional and case-control studies in surgery (STROCSS) criteria^25^. Supplemental Digital Content 1, http://links.lww.com/JS9/A529.

### Emergency medical system and master plan for the established trauma system in South Korea

In South Korea, the current emergency medical system was established in the 2000s based on the promulgation of the Emergency Medical Service Act in 1994^21^. The system was created by rating the following three levels of EMIs according to the level of available resources: regional emergency medical center (REMC), local emergency medical center (LEMC), and local emergency medical institution (LEMI). The REMC is the highest level of EMI. In 2019, there were 38 REMCs, 124 LEMCs, and 240 LEMIs, which received financial support of 250 million USD from the government^12,26^.

The Korean government and the medical society created a master plan for the national trauma system establishment in 2012^21,27,28^. The law on the establishment and operation of RTCs was enacted in the Emergency Medical Service Act and the candidate RTCs were recruited among REMCs and LEMC with greater than or equal to 500 beds that met the criteria, and selected after evaluation. Essentially, each institution should be equipped with facilities including two or more resuscitation rooms, one or more operation room for emergency surgeries, and an intensive care unit with greater than or equal to 20 beds dedicated to trauma patients. Sixty-seven million USD was provided per institution for the construction of these, and labor cost for the 25 dedicated trauma doctors per institution has been fully supported since designation. Seventeen RTCs were designated and nine of them were officially opened by 2019^12,26,28^.

### Outcome measures

**Figure 1:**
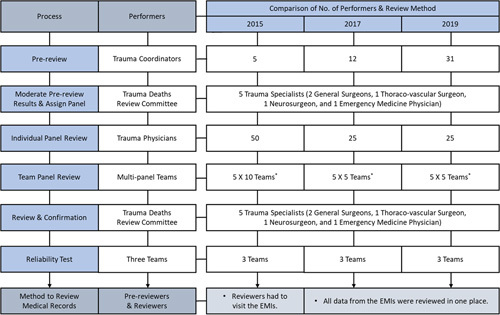
Process of the Multipanel Review for Preventable Trauma Death Rate. ^*^One team comprised two general surgeons, one thoraco-vascular surgeon, one neurosurgeon, and one emergency medicine physician. EMIs, emergency medical institutions.

Our primary outcome measure was in-hospital trauma mortality, except in the case of patients coded as deaths on arrival and those who had arrived with no vital signs and died in the emergency department. Postdischarge deaths were not included because the data were not available in the NEDIS database. We evaluated and compared the in-hospital trauma mortality in two ways:

First, the PTDR was investigated through a multipanel review process for death cases selected by statistical sampling. The investigation was conducted in the following order: survey design, extraction of the sample population, data collection, panel review, reliability test for the review process, and result analysis according to the WHO guidelines for trauma quality improvement program^29^. We used a structured review form including audit filters for the review of medical records (SDC, Figure A.1, Supplemental Digital Content 2, http://links.lww.com/JS9/A530). The review form was based on the data sheet for use in preventable death panel reviews with embedded audit filters as in the WHO guidelines. Designated prereviewers comprising trauma coordinators working in RTCs investigated and recorded the general characteristics of patients and injury-, transport-, and treatment-related information before the panel review. The preventable death panel comprising dedicated trauma professionals performed an initial evaluation of the preventability of deaths using the data extracted by the prereviewers. For a multidisciplinary review, five to ten teams were formed, with each team comprising two general surgeons, one thoracic surgeon, one neurosurgeon, and one emergency physician. After individual evaluations, the panels from each team came together to present the cases they had reviewed, and they discussed and reached a consensus for the final assessment of the preventability of trauma-related deaths. Besides, a committee comprising five trauma specialties was responsible for developing the guidelines for the whole review process. When preventability was not decided by the multipanel review, the committee reviewed and confirmed the final decisions. To evaluate the reliability of the panel review, three teams were selected. They repeated reviews for 4–5% of the overall cases that had already been reviewed by other teams (Fig. 1). Due to the limitations of the methodology that takes a considerable amount of time for sampling and the multipanel review process, we performed a national survey on PTDR every 2 years. The total sample size initially targeted was 1000 in 2015, 1300 in 2017, and 1300 in 2019. After reviewing the sample size according to the level of target error, but considering the cases to be excluded from the panel review, the survey sample size was determined to be 1131, 1862, and 1692, respectively. It was estimated that the target sample size would be able to meet the limit of error of ~±4.5%p in 2015, ±3.8%p in 2017, and ±3.3%p in 2019 at 95% confidence levels for estimation of the population ratio (SDC, Table A.1, Supplemental Digital Content 2, http://links.lww.com/JS9/A530). The PTDR of the entire population was calculated by applying weights to the proportion of definitive preventable and potentially preventable cases among the total number of trauma deaths for which the review was completed. Factors responsible for preventable trauma deaths were identified and PTDR were compared statistically by year.

Second, the RATM prediction model was based on findings from the literature review, expert opinion, and our previous work on the evaluation of trauma outcomes after the establishment of a national trauma system in South Korea^28^. We constructed an RATM prediction model using the extended-International Classification of Disease Injury Severity Score (ICISS). The ICISS took an empirical estimation approach for injury severity scoring with the International Classification of Disease (ICD) survival probability formulation. The probability of survival (Ps) was an ICD code-specific estimate of the survival probability associated with a particular injury. Conventionally, the ICISS is calculated as the product of Ps for as many as 10 injuries and ranges from 0 (unsurvivable) to 1 (null probability of death)^30,31^. The extended-ICISS is adjusted for age and the Revised Trauma Score (RTS) in addition to the ICISS. To calculate the RTS, the initial physiologic parameters (Glasgow coma score, respiratory rate, and systolic blood pressure) at emergency department admission were used. Using the RATM prediction model, we evaluated the extent of improvement in the trauma mortality rates after the trauma system was established by comparing the risk-adjusted mortality according to Ps from the NEDIS between 2015 and 2019.

### Statistical analysis

To calculate the national PTDR estimate and improve the efficiency of the panel review, we selected cases for trauma death review through stratified sampling. The stratification was designed by sex, age, place of death, type of emergency institution, and geographical (region) variables. To estimate the population PTDR, the sample hospital level and death weights were calculated according to the sample design method and applied to analyze the sample-designed survey data (SDC, Table A.1, Supplemental Digital Content 2, http://links.lww.com/JS9/A530). In the PTDR panel review data, characteristics of patients with preventable trauma death outcomes in 2015, 2017, and 2019 were compared using the *χ* ^2^ -test. A mixed-effects model with a logit link function was used to compare preventable death outcomes between years, and odds ratios and their 95% CIs were calculated. To account for within- and between-region variations, we used region-specific random intercepts, while potential confounding factors (stratifying variables such as sex, age, place of death, and type of emergency institution) were adjusted for. We evaluated the performance of the mixed-effects logistic regression model using the Hosmer–Lemeshow goodness-of-fit test and area under the curve (AUC) statistic. The agreement between the panel teams was evaluated using Cohen’s kappa index. To construct the RATM prediction model using the NEDIS data, we fitted a logistic regression utilizing the variables of ICISS, age, and RTS; patients with missing data for these variables were excluded. Youden’s index was used to predict the outcome of death. To test the trend of death rates over the years, we conducted a Cochran–Armitage trend test. All statistical analyses were performed using R (Version 4.0.3), and two-sided *P* values <0.05 indicated statistical significance.

## Results

### Preventable trauma death rate

**Figure 2:**
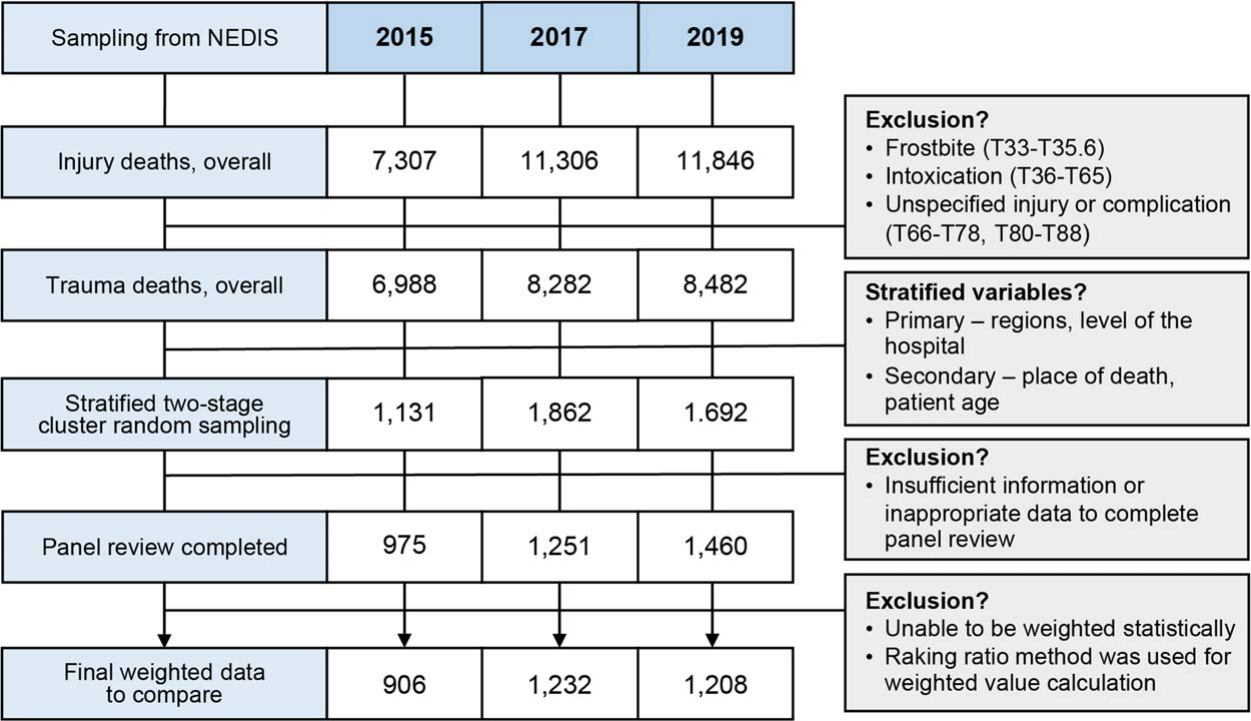
The flow chart for the comparative analysis of the preventable trauma death rates between 2015, 2017, and 2019 based on the multipanel review. NEDIS, National Emergency Department Information System.

In the NEDIS, the total number of trauma deaths in 2015, 2017, and 2019 were 6988, 8282, and 8482, respectively (Fig. 2). Of these, the number of sample cases included in the PTDR review was 975 (14.0%), 1251 (15.1%), and 1460 (17.2%), respectively; the number of final weighted data for comparative analysis was 906, 1232, and 1208, respectively (Table 1). In the reliability test of the panel review, the kappa index was 0.49 in 2015, 0.61 in 2017, and 0.40 in 2019, indicating moderate, substantial, and moderate agreements, respectively.

**Table 1:** Basic characteristics of included patients by preventable trauma death rates across study years^*^.

	Number of trauma deaths (%)			Number of preventable trauma deaths (%)			*P*	
Variable	2015	2017	2019	2015	2017	2019	2015 vs. 2019	2017 vs. 2019
Total	906	1232	1208	276 (30.5)	245 (19.9)	190 (15.7)	<0.001	<0.001
Sex								
Male	628	868	845	186 (29.6)	172 (19.8)	138 (16.3)	<0.001	0.07
Female	278	364	364	91 (32.6)	73 (20.0)	51 (14.3)	<0.001	0.04
Age, years								
≤ 14	16	17	16	2 (12.5)	3 (17.6)	1 (6.3)	1.00	0.64
15 – 54	263	351	372	66 (25.2)	56 (15.9)	33 (8.9)	<0.001	0.01
≥ 55	627	863	821	208 (33.2)	186 (21.5)	157 (19.1)	<0.001	0.24
Place of death								
DOA at the first hospital	164	284	381	7 (4.3)	4 (1.4)	3 (0.8)	0.02	0.70
At the ED of the first hospital	100	123	97	33 (33.0)	46 (37.4)	31 (32.0)	1.00	0.49
After hospitalization at the first hospital	395	507	462	146 (37.1)	92 (18.2)	81 (17.5)	<0.001	0.87
Transfer to the second or additional hospitals	247	318	267	90 (36.3)	102 (32.0)	75 (28.1)	0.05	0.34
Type of emergency medical institution								
RTC	223	383	542	46 (20.7)	73 (19.2)	76 (14.0)	0.03	0.05
REMC	74	232	173	25 (33.8)	55 (23.7)	41 (23.7)	0.14	0.34
LEMC	481	485	363	166 (34.4)	106 (22.0)	47 (12.9)	<0.001	0.001
LEMI	128	135	121	39 (30.5)	11 (8.0)	23 (19.0)	0.05	0.02
Type of injury								
Blunt	880	1190	1164	269 (30.6)	239 (20.1)	188 (16.2)	<0.001	0.001
Penetrating	6	12	16	1 (14.3)	2 (16.7)	2 (12.5)	1.000	1.000
Other/unknown	20	29	28	6 (30.8)	3 (10.3)	0 (0.0)	<0.001	<0.001
Transfer from another hospital^†^								
No	612	902	940	174 (28.4)	140 (15.5)	115 (12.2)	<0.001	0.05
Yes	249	330	268	85 (34.1)	105 (31.8)	75 (28.0)	0.16	0.35

* All data are shown as numbers (percentages) and all values are weighted.

† There were 45 patients with unknown transfer status in 2015.

DOA, death on arrival; ED, emergency department; LEMC, local emergency medical center; LEMI, local emergency medical institution; REMC, regional emergency medical center; RTC, regional trauma center.

**Figure 3:**
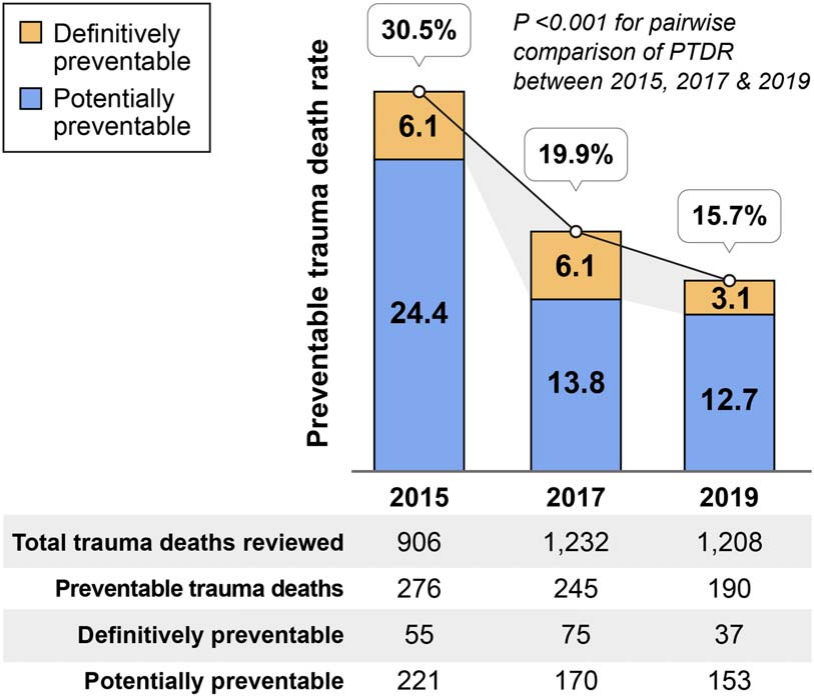
Comparison of the preventable trauma death rates between 2015, 2017, and 2019 in South Korea. PTDR, preventable trauma death rate.

The PTDR in 2019 was lower than that in both 2015 (15.7 vs. 30.5%, *P*<0.001) and 2017 (15.7 vs. 19.9%, *P*<0.001) (Fig. 3). Although the PTDR in the two place of death subgroups, death on arrival (DOA) at the first hospital and after hospitalization decreased significantly from 2015 to 2019, and no significant difference in PTDR was observed in terms of the place of death from 2017 to 2019 (Table 1). Regarding the hospitalization in EMIs, RTC, LEMC, and LEMI presented significantly lower PTDRs in 2019. PTDR significantly decreased in the no-transfer group from 2015 (28.4 vs. 12.2%, *P*<0.001) and 2017 (15.5 vs. 12.2%, *P*=0.05) to 2019. Overall, the PTDRs of many subgroups significantly decreased from 2015 to 2019, while some became statistically insignificant between 2017 and 2019.

**Figure 4:**
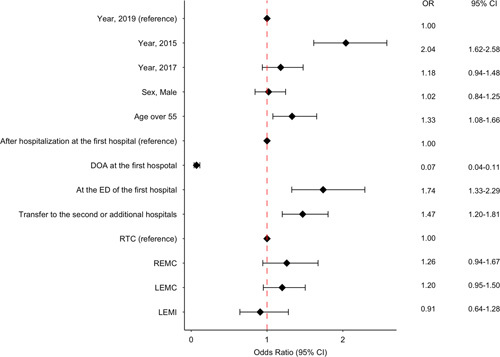
Multivariable analysis of the risk factors for preventable trauma deaths. DOA, death on arrival; ED, emergency department; LEMC, local emergency medical center; LEMI, local emergency medical institution; OR, odds ratio; RTC, regional trauma center; REMC, regional emergency medical center.

In the most valid logistic regression model for PTDR versus non-PTDR outcomes (*P*=0.45, Hosmer–Lemeshow test, AUC=0.712), year (2015 vs. 2019), age (over 55 years vs. not), and place of death were significant factors influencing preventable death. In the model adjusted for potential confounders that were used for stratified sampling in the 2019 PTDR review, the risk of preventable death indicated a significantly higher OR of 2.04 (95% CI=1.62–2.58) in 2015 than in 2019, while the risk of preventable death from 2017 to 2019 was not statistically significant (OR=1.18, 95% CI=0.94–1.48) (Fig. 4).

### Risk-adjusted trauma mortality

**Figure 5:**
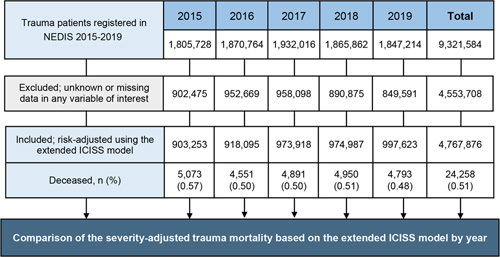
The flow chart for the comparative analysis of the trauma outcomes from 2015 to 2019 using the risk-adjusted trauma mortality prediction model based on the extended-ICISS model. ICISS, International Classification of Disease Injury Severity Score; NEDIS, National Emergency Department Information System.

A total of 9 321 584 patients with trauma were enrolled between 2015 and 2019. After excluding patients with missing variable(s) in the extended-ICISS, the total number of trauma patients and deaths between 2015 and 2019 was 4 767 876 and 24 258 (0.51%), respectively (Fig. 5). Of these, the overall death rates for patients with trauma who had Ps less than 0.25, Ps=0.25–0.75, and Ps greater than 0.75 were 71.99, 34.06, and 0.36%, respectively.

**Figure 6:**
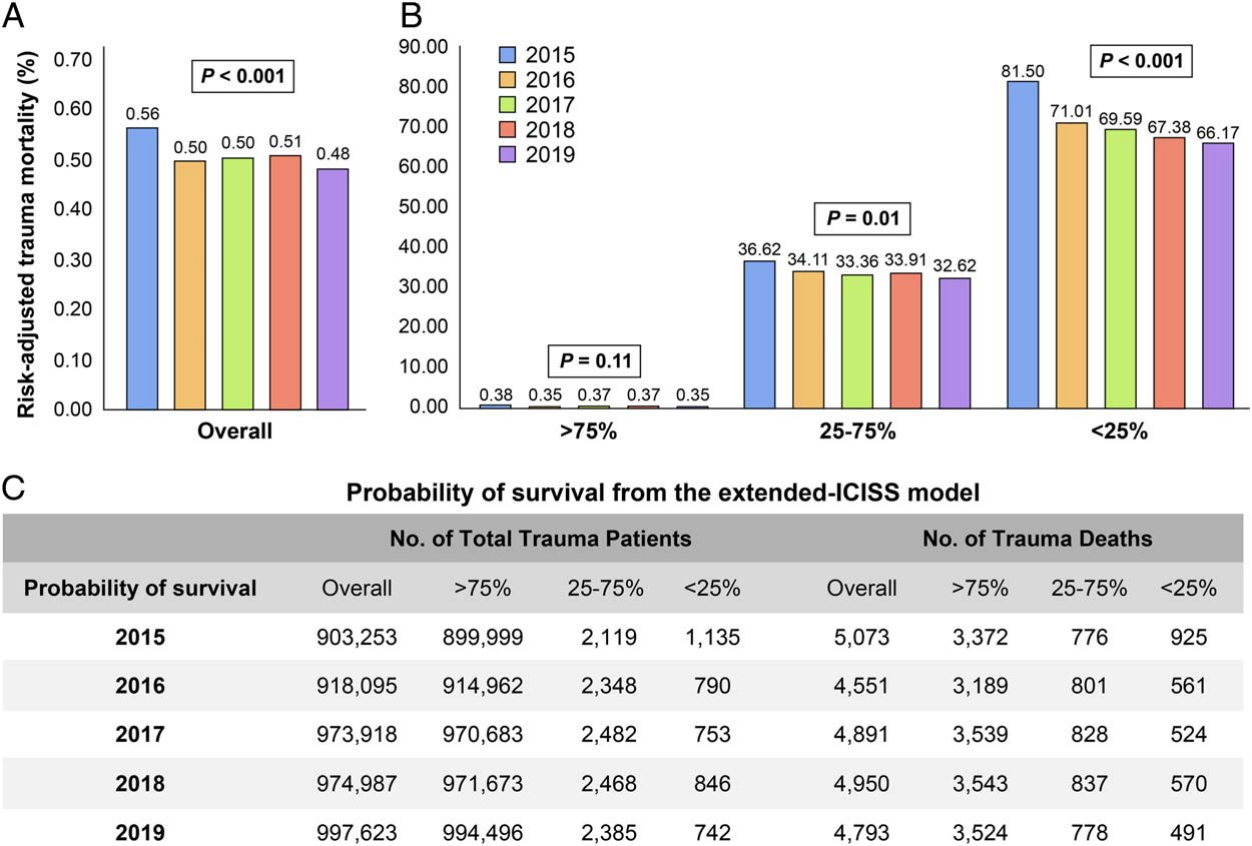
Comparison^*^ of the risk-adjusted trauma mortality using the extended-ICISS model from 2015 to 2019 in South Korea; (A) risk-adjusted trauma mortality in the overall population, (B) risk-adjusted trauma mortality according to the probability of survival, and (C) number of total trauma patients and deaths over years. ^*^ *P* values were obtained from the Cochran–Armitage trend test under the hypothesis $H_{0}:\;$ no trend versus $H_{1}$ : decreasing trend over the years. ICISS, International Classification of Disease Injury Severity Score.

Using data of the patients with trauma and no missing data in the NEDIS, we developed the RATM prediction model (SDC, Table A.2, Supplemental Digital Content 2, http://links.lww.com/JS9/A530). The model was statistically considered outstanding, with an AUC of 0.955 (95% CI; 0.953–0.956) (SDC, Figure A.2, Supplemental Digital Content 2, http://links.lww.com/JS9/A530). In the model, the total trauma mortality was the highest in 2015 at 0.56%, followed by that in 2016 and 2017 (0.50%), 2018 (0.51%), and 2019 (0.48%) (Fig. 6A). The Cochran–Armitage trend test revealed a significant decrease in mortality over the years (*P*<0.001). In the RATM model, the number of deaths for more severe patients (i.e. Ps<0.25) significantly decreased from 81.50% in 2015 to 66.17% in 2019 (*P*<0.001), while the decrease in the death rate for patients with trauma (Ps>0.75) was not statistically significant (*P*=0.11). The mortality of patients with Ps=0.25–0.75 was moderately decreased (*P*=0.01) (Fig. 6B). We have presented the numbers of total trauma patients and deaths over the years according to the Ps in Figure 6C.

**Figure 7:**
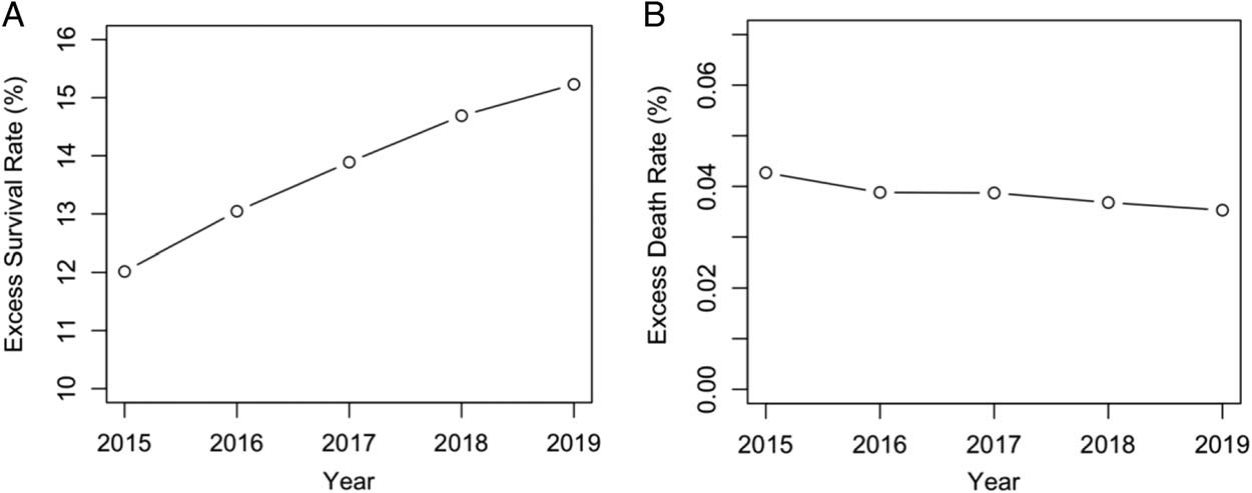
Trend^*^ of the excess survival rate (A) and excess death rate (B) of patients with trauma between 2015 and 2019 in South Korea based on the extended-ICISS model. ^*^Youden’s index was used as the cut-off value.

Based on the RATM prediction model, we investigated the trends of the prediction of excess survival rates (actual survivors/prediction of death resulting from the model) (Fig. 7A) and excess death rates (actual deaths/prediction of survival) (Fig. 7B). Note that we used the Youden’s index to obtain the predicted death outcome. The excess survival rate increased over the years (e.g. from 12.02 in 2015 to 15.23% in 2019), while the excess death rate decreased (e.g. from 0.043 to 0.035%).

## Discussion

In this nationwide follow-up observational study based on a national survey of PTDR and RATM, we found that trauma outcomes improved significantly during the study period owing to the establishment of a trauma system in South Korea. We observed a 14.8% reduction in PTDR during the study period, representing 1247 additional lives saved in 2019 compared to that in 2015. The RATM decreased by 0.08%, representing nearly 800 additional lives saved. In particular, the decrease was the greatest in the more severe patient group with Ps less than 25%. From these results, we confirm that the original goal of the trauma system in South Korea to reduce the trauma mortality rate by concentrating the severe patients in RTCs has been achieved^25,28,32^.

In order to evaluate the effectiveness of the established trauma system in South Korea, we first conducted a national survey of PTDR using the multipanel review. Originally, the goal of implementing the national trauma system in South Korea was to reduce the PTDR, which was higher by greater than 35% in a previous survey^16^. The original goal was to achieve less than 20% by 2020, but based on our study results, it had decreased to 15%, thus exceeding the goal. The panel review methodology has been criticized because the preventability assessment may be subjective and there may be variations in reliability between different panel assessments^33–35^. Nonetheless, death panel reviews remain a straightforward method of accomplishing the goal of assessing and improving the quality of care. The WHO trauma quality improvement program guidelines state that the starting point for building quality improvement in trauma care is the identification of preventable trauma deaths based on data collection and monitoring^29^. Despite the limitations in the methodology, the PTDR survey was undoubtedly a necessary process in South Korea, where the implementation of an inclusive national trauma system was just begun. Moreover, for a fair comparison of PTDR before and after the trauma system implementation, we followed the same methodology that was used previously^14–16^. Previous PTDR surveys were conducted sporadically on a small scale, but we applied the same review process serially and repeatedly using large-scale data from EMIs nationwide^28,32^. We observed a moderate to substantial agreement in the reliability test for the panel review in the three follow-up surveys. We believe that the reliability of our results, especially the decreasing trend of PTDR, has been validated.

Measuring the effect of an established national trauma system using the RATM prediction model supplements the limitations of the panel review method. Compared to the PTDR survey, the RATM method was able to create a statistically valid mortality model based on a large amount of data from all trauma populations who visited EMIs nationwide. Although risk adjustment is essential to measure and compare trauma outcomes, many recent studies have not provided high-quality evidence due to the difficulty of risk adjustment in trauma populations with various characteristics^10^. Considering the situation in South Korea, where most EMIs do not routinely perform Abbreviated Injury Scale coding, we devised an ICISS model based on ICD codes. Furthermore, we extended the model by adding RTS and age, which reflect the physiological parameters and underlying conditions of patients with trauma. The extended-ICISS model we devised was confirmed to be statistically appropriate, and we were able to confirm the effect of establishing the national trauma system in South Korea through comparative analysis based on the model. Overall, trauma mortality showed a tendency to decrease, and a significant improvement was observed among severe cases with Ps less than 25%. We observed that patients with severe trauma were concentrated in RTCs after national trauma system establishment in South Korea in another previous study^28^. Considering these results, it is reasonable to assume that the effort to establish a national trauma system based on the implementation of RTCs has contributed the most to the reduction in mortality of patients with highly severe trauma.

Several studies and reports have indicated that trauma system establishment improves outcomes, especially that establishing a regional trauma system based on trauma centers is a shortcut to lower trauma mortality^5,9,36–40^. In the United States, trauma systems based on trauma center implementation have been established since the 1970s^41,42^. This suggests that the center at the highest level plays the role of a leader in building a regional trauma system^43–45^. Recent studies conducted in Canada have also reported a reduction in trauma mortality rate through the establishment of a national trauma system^9,37,39^. Moore *et al*. reported that regions with better trauma system components had better survival rates. Through the establishment of the trauma system, there was an 18.2% relative decrease in risk-adjusted mortality in level I or II trauma centers over 6 years, which is expected to lead to a reduction in the burden of injury for the whole country^37^. However, since most previous studies were mainly conducted in HICs where the health care system and emergency medical system are well established, LMICs that have just started to implement a trauma system may have insufficient information to guide its implementation. Although several studies conducted in Asia and other LMICs were included in a recent systematic review published in 2018 on the impact of trauma system structure^10^, most studies in the quantitative meta-analysis were from the United States and European countries. In our study, however, we observed that the trauma outcome improved significantly in the short time following the establishment of the national trauma system through government-led efforts, despite South Korea’s trauma system being at the level of LMICs until the early 2010s.

Because trauma is the most time-sensitive condition and its occurrence is difficult to predict compared to other disease categories in emergency medical care, a multidisciplinary simultaneous approach for optimal care is essential. However, there are limitations to establishing countermeasures by an individual or institution in a medical environment in a capitalist market economy because more resources are required instantaneously in trauma care. Although South Korea started building trauma systems 10 years ago, which is considered late compared to other developed countries, this study showed that such efforts achieved the goal of reducing PTDR and RATM in a short time. It is meaningful that this study was able to numerically evaluate the effects of the government-led national trauma system establishment project, including financial support from public funds. This study could be a good model for many LMICs with a high burden of injury owing to the lack of a trauma system.

This study has several limitations. First, the retrospective study design based on the data from a known registry precluded the analysis of unrecorded factors or missing values. Especially, in the process of making the RATM model, nearly half of the values were not available for variables that we could analyze, because the initial physiologic parameters were not registered at the LEMIs. Nonetheless, the risk-adjusted model was statistically valid because we obtained a sufficient number of cases even though missing values were excluded. Second, there were some differences in the characteristics of the samples extracted for each period in 2015, 2017, and 2019. Although sampling for the three periods was conducted similarly; however, since the collection of the 2019 death cases proceeded during the peak of the coronavirus disease 2019 outbreak (in 2021), data from some medical institutions were insufficiently collected. Moreover, with respect to these limitations, we referred to previous studies^9,10,29,30^ and tried to construct an adjusted logistic regression model by identifying and imputing confounding factors, but it was difficult to overcome the limitations completely. Especially, the influence of DOA patients could not be completely excluded. Third, the NEDIS is not completely population-based. However, because almost all trauma patients, especially those with severe trauma, are expected to visit EMIs within the South Korea’s medical environment, with good accessibility to the database, suggests a representative trauma population in the country. Fourth, the evaluation of preventability relied entirely on panel review, which has limitations in objective reproducibility. Fifth, there was a time lag between the master plan development, at the end of 2012, and the actual implementation of the national trauma system, and we only focused on the time points since 2015. We believe it will take a 2-year interval to allow the emergency medical system and the EMIs to adjust to its new policies and implement new systems.

In conclusion, we observed significant reductions in PTDR and RATM during the 5-year follow-up period since the establishment of the national trauma system in 2015 in Korea. Although there are limitations in generalizing the results of this study, which evaluated the effectiveness of the national trauma system, to other countries with different medical environments, it could be a good model for LMICs with a large burden of injury since their trauma system is not yet well established.

## Ethical approval

This study was approved by the Institutional Review Board of Ajou University (AJIRBMED-EXP-20-473).

## Sources of funding

This study was funded by the Ministry of Health and Welfare, Republic of Korea (2020-10-16). The funder of the study had no role in the study design, data collection, data analysis, data interpretation, or writing of the manuscript. All authors had full access to all data in the study and had final responsibility for the decision to submit for publication.

## Author contribution

J.K. and M.L.: conceptualization, methodology, formal analysis, investigation, data curation, writing – original draft; Y.K.: methodology, writing – review and editing; J.M. and Y.H.: investigation, data curation, writing – review and editing; S.S. and S.K.: formal analysis, investigation, data curation, writing – review and editing; J.K.: resources, data curation, writing – review and editing; K.J.: conceptualization, methodology, data curation, writing – review and editing, supervision, project administration, funding acquisition.

## Conflicts of interest disclosure

The authors declare that they have no conflicts of interest.

## Research registration unique identifying number (UIN)

1. Name of the registry: Clinical Research Information Service (CRIS). The CRIS is a non-profit online registration system for clinical trials (researches) to be conducted in South Korea. It has been established at the Korea Disease Control and Prevention Agency with support from the Ministry of Health and Welfare. It joined the WHO International Clinical Trials Registry Platform as 11th member of Primary Registry.
2. Unique Identifying number or registration ID: KCT0008031.
3. Hyperlink to our specific registration (must be publicly accessible and will be checked): https://cris.nih.go.kr/cris/search/detailSearch.do/23697
.

## Guarantor

Kyoungwon Jung, MD, PhD, Division of Trauma Surgery, Department of Surgery, Ajou University School of Medicine, 164 World cup-ro, Yeongtong-gu, Suwon 16499, Korea. Tel: +82 312 197 491, fax: +82 312 197 781, E-mail: jake98@daum.net, jake98@ajou.ac.kr.

## Data availability statement

The data that support the study findings are available upon reasonable request from the corresponding author.

## Supplementary Material
