# Supplementary material for: Trauma system establishment and outcome improvement: a retrospective national cohort study in South Korea
Source: Int J Surg. 2023 May 18;109(8):2293–302. doi: 10.1097/JS9.0000000000000481 (PMC10442102; doi:10.1097/JS9.0000000000000481)
Supplement: Supplementary file 2 [file js9-109-2293-s002.docx]

**Supplemental Digital Content (SDC)**

**Figure A.1.** The Structured Review Form Including Audit Filters for the Multi-Panel Review

**
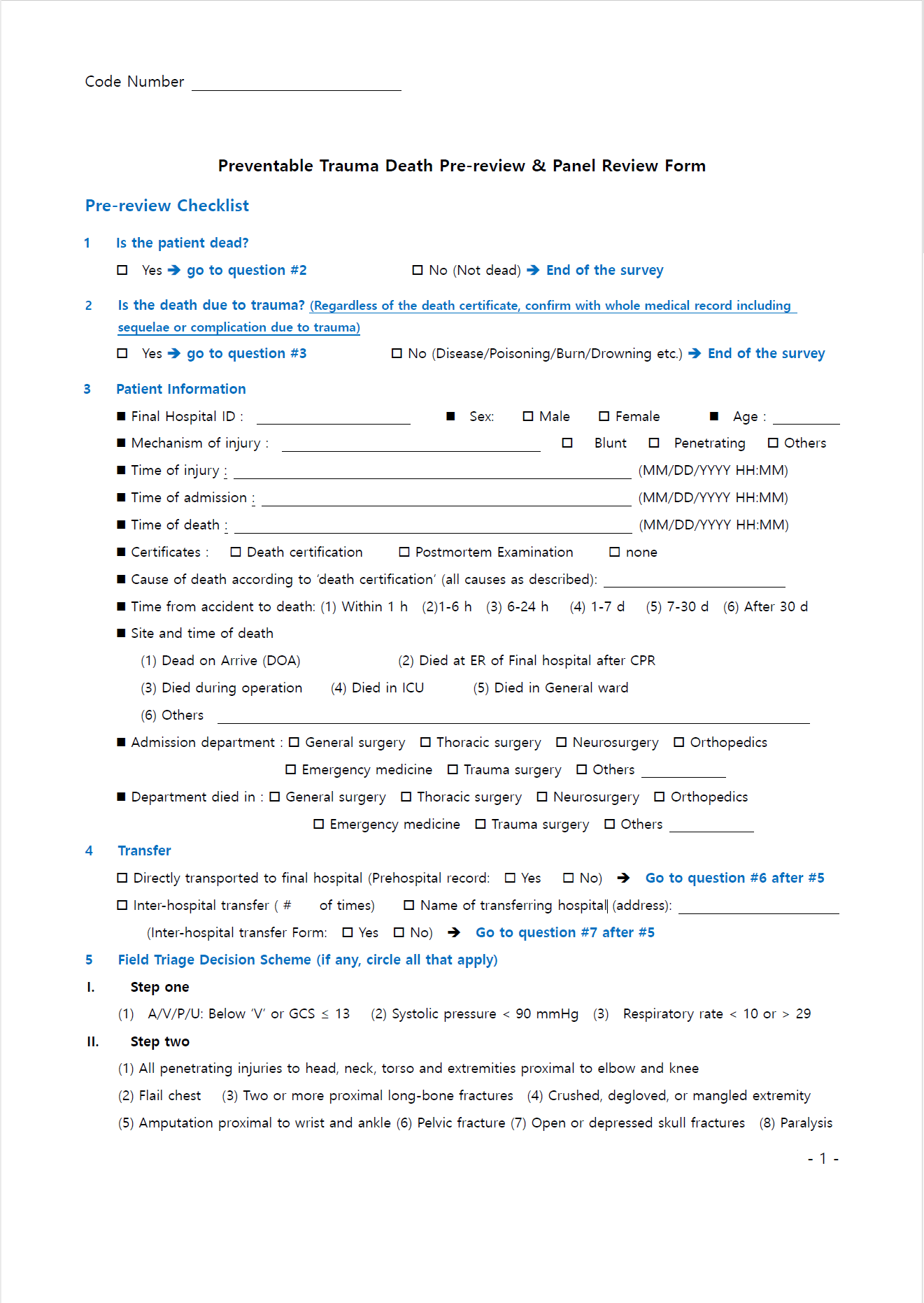
**

**Figure A.1.** (Continued)

**
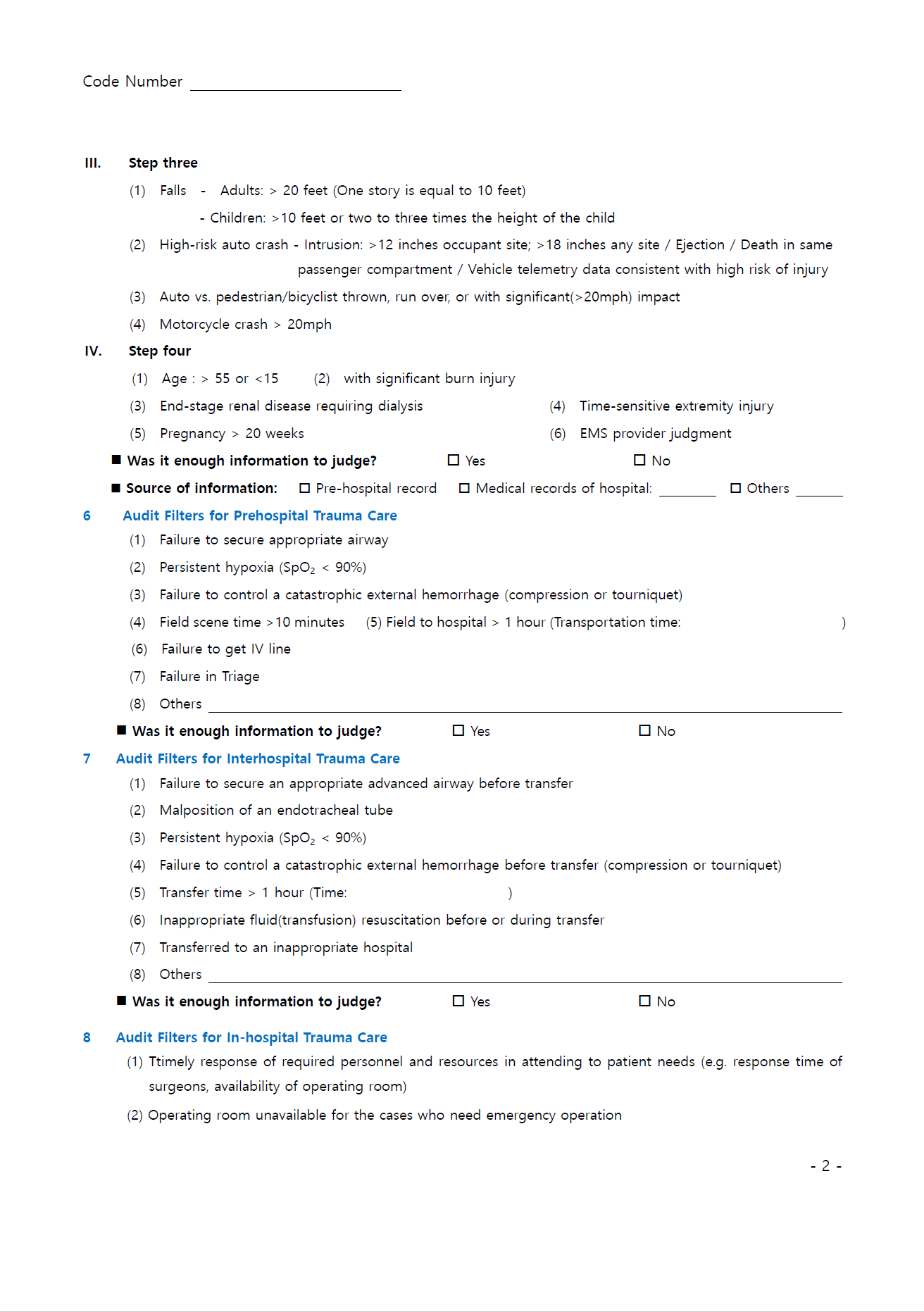
**

**Figure A.1.** (Continued)

**
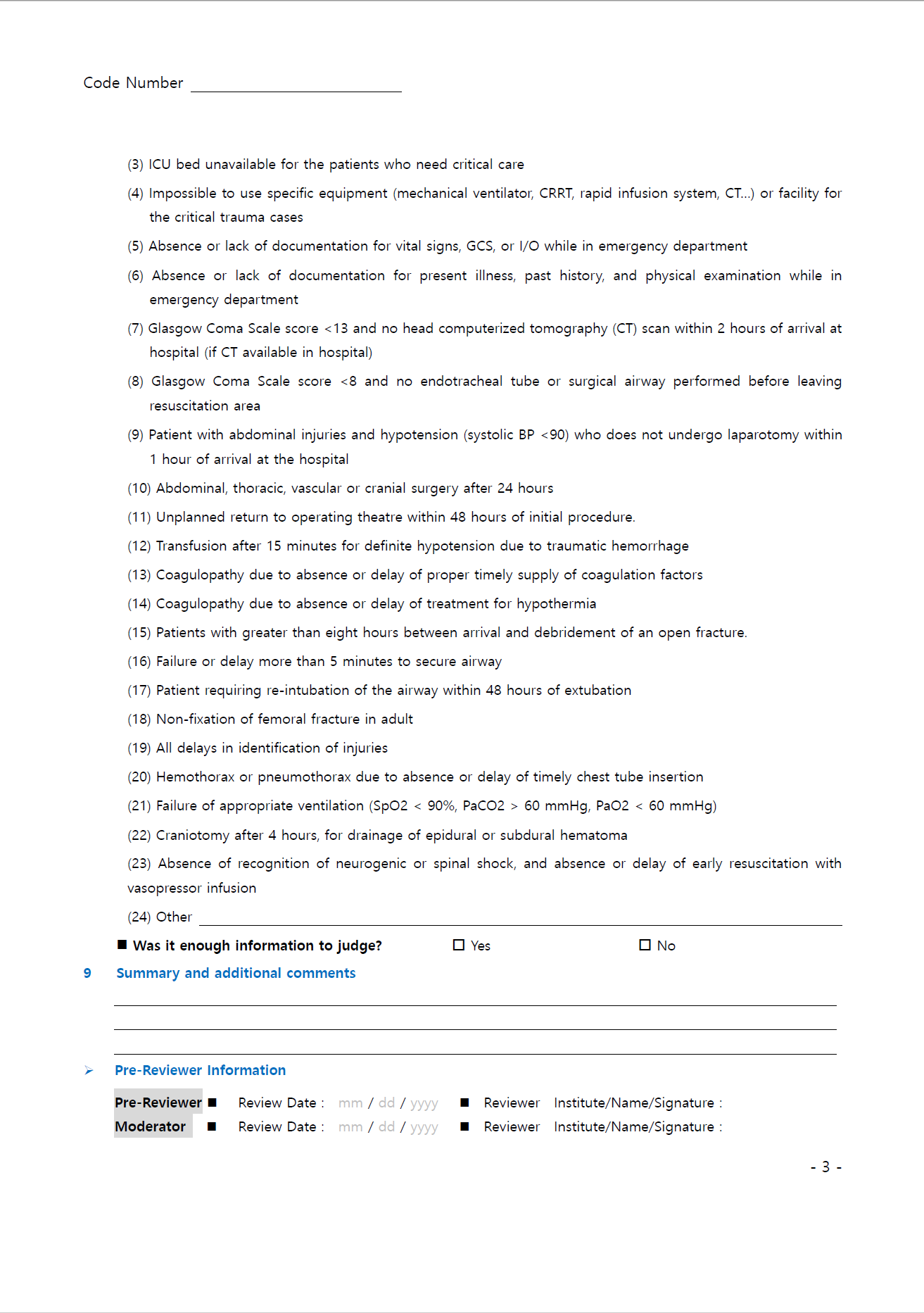
**

**Figure A.1.** (Continued)

**
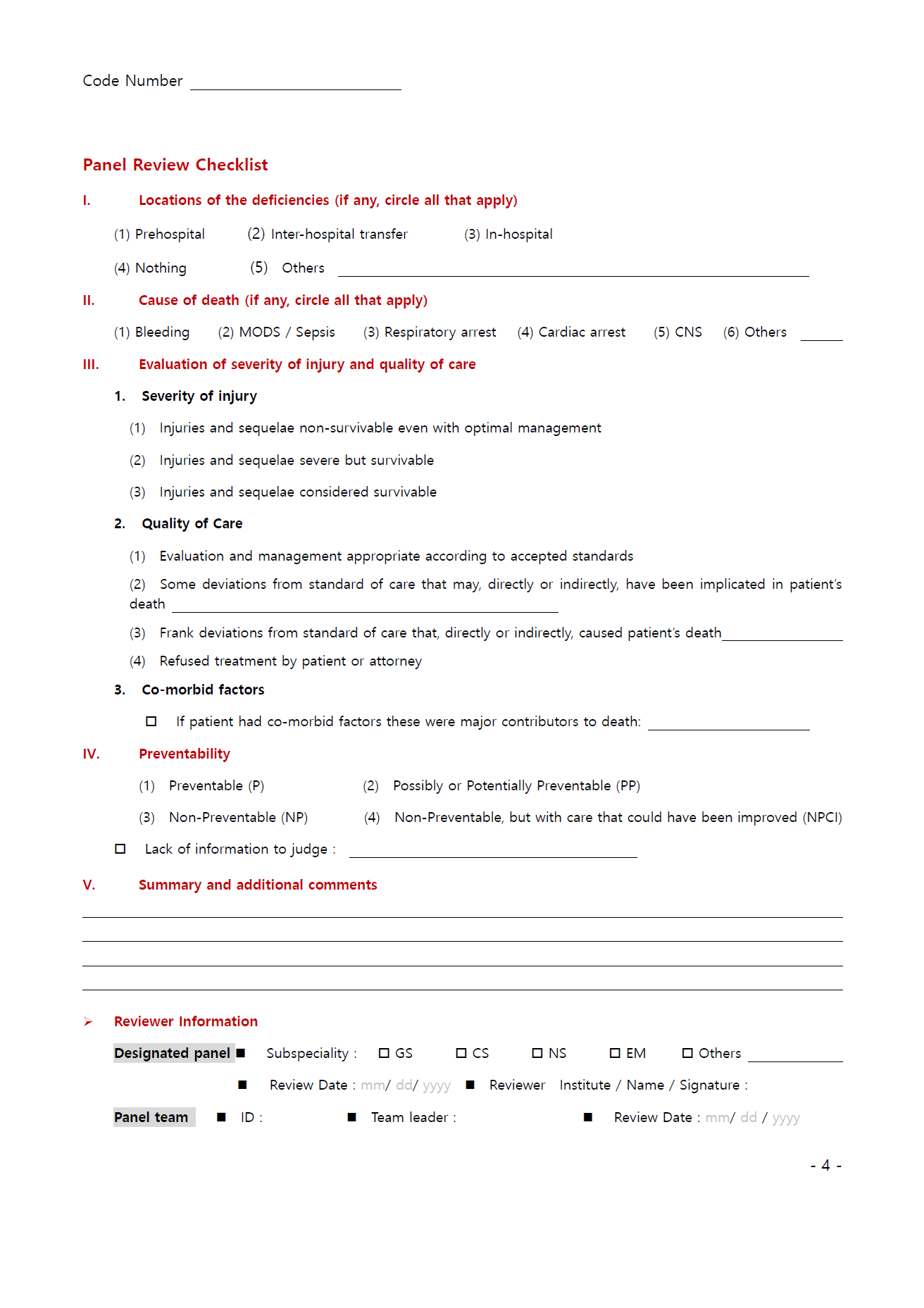
**

**Table A.1.** Summary of Method for Sampling and Estimation of Traumatic Deaths.

| Item | Detailed Method | |
| --- | --- | --- |
| Population | Target Population | ▪ Trauma deaths who were transferred to the emergency medical institutions in South Korea |
|  | Investigation Population | ▪ Trauma deaths in emergency medical institutions which have more than one trauma death per year |
| Sampling Unit | Primary Sampling Unit | ▪ Emergency medical institutions |
|  | Secondary Sampling Unit | ▪ Trauma deaths in emergency medical institutions |
| Stratification | First Stratification Variable  (Stratification of Emergency Medical Institutions) | ▪ Region (5): Seoul, Incheon/Gyeonggi, Daejeon/Chungcheong/Gangwon, Gwangju/Jeolla/Jeju, Busan/Daegu/Ulsan/Gyeongsang  ▪ Level (type) of emergency medical institutions (3): REMC/RTC, LEMC, LEMI  ▪ Number of trauma deaths (3): ≤9, 10–29, ≥30 |
|  | Second Stratification Variable  (Stratification of Death) | ▪ Place (timing) of trauma deaths (3): DOA, At emergency department, After hospitalisation  ▪ Patients’ age (3): ≤14, 15–54, ≥55 |
| Sample Size | ▪ The total sample size we initially targeted was 1,000 in 2015, 1,300 in 2017, and 1,300 in 2019 after reviewing the sample size according to the level of target error, but considering the cases to be excluded from the panel review, the survey sample size was determined to be 1,131, 1,862, and 1692.  ▪ The target sample size was expected that stable estimation would be possible to meet the limit of error of approximately ±4.5%p in 2015, ±3.8%p in 2017, and ±3.3%p in 2019 at 95% confidence levels for the estimate of the population ratio. | |
| Sampling | ▪ The sample emergency medical institutions were extracted by stratified two-stage cluster random sampling.  ▪ The sample trauma deaths were extracted by stratified random sampling, totalling 1,131 in 2015, 1,862 in 2017, and 1,692 in 2019. | |
| Calculation of  Sample Weight | ▪ To estimate PTDR of the population, the sample weight of each hospital level and death was calculated according to the sample design method and applied to analyse the sample-designed survey data. | |
| Estimation | ▪ Calculation of the parameter of interest (preventable trauma death rate) by the weighted estimates using weights.  ▪ Calculation of the standard error and the margin of error for the estimate of the parameter of interest (preventable trauma death rate). | |

REMC, Regional Emergency Medical Center; RTC, Regional Trauma Center; LEMC, Local Emergency Medical Center; LEMI, Local Emergency Medical Institution; DOA, death on arrival.

**Table A.2.** Extended-ICISS Model for Death Outcome^*^.

| Variables | OR | 95% CI | P value |
| --- | --- | --- | --- |
| ICISS | 0.001 | 0.001, 0.002 | <0.001 |
| Age <=55  Age >55 | 1.000  6.331 | 6.098, 6.573 | <0.001 |
| RTS | 0.309 | 0.305, 0.312 | <0.001 |

^*^Logistic regression was used.

OR, odds ratio; CI, confidence interval; ICISS, International Classification of Disease Injury Severity Score; RTS, Revised Trauma Score.

**Figure A.2.** ROC Curve^*^ of the Extended-ICISS Model.


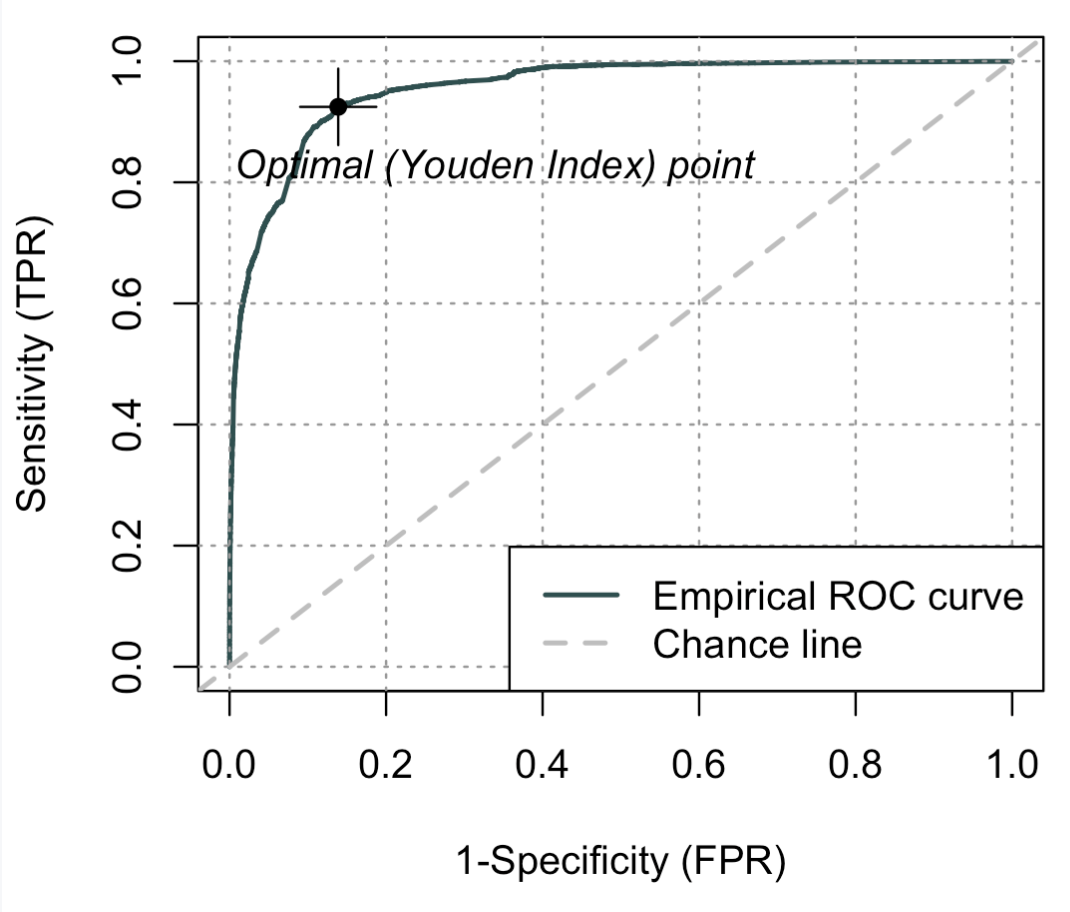


^*^AUC was 0.955 (95% CI: 0.953 – 0.956).

ROC, receiver operating characteristic; ICISS, International Classification of Disease Injury Severity Score; AUC, area under curve.
